# Drivers of molecular and morphometric variation in *Triatoma brasiliensis* (Hemiptera: Triatominae): the resolution of geometric morphometrics for populational structuring on a microgeographical scale

**DOI:** 10.1186/s13071-020-04340-7

**Published:** 2020-09-07

**Authors:** Edgard H. Kamimura, Maria Carolina Viana, Maurício Lilioso, Fernanda H. M. Fontes, Dayane Pires-Silva, Carolina Valença-Barbosa, Ana L. Carbajal-de-la-Fuente, Elaine Folly-Ramos, Vera N. Solferin, Patricia J. Thyssen, Jane Costa, Carlos E. Almeida

**Affiliations:** 1 Instituto de Biologia, Universidade Estadual de Campinas (UNICAMP), Campinas, Brazil; 2 Centro Nacional de Diagnóstico e Investigación en Endemo-Epidemias (CeNDIE), Administración Nacional de Laboratorios e Institutos de Salud “Dr. Carlos Malbrán” (ANLIS), Buenos Aires, Argentina; 3 Consejo Nacional de Investigaciones Científicas y Técnicas, Buenos Aires, Argentina; 4 Universidade Federal da Paraíba (UFPB), Campus IV, Rio Tinto, Brazil; 5 Laboratório de Biodiversidade Entomológica, Instituto Oswaldo Cruz, Fundação Oswaldo Cruz (IOC/Fiocruz-RJ), Rio de Janeiro, Brazil

**Keywords:** Triatomine ecology, Population structure, Phenotypic plasticity, Chagas disease, Vectors

## Abstract

**Background:**

The protozoan *Trypanosoma cruzi* circulates in semiarid areas of northeastern Brazil in distinct ecotopes (sylvatic, peridomestic and domestic) where *Triatoma brasiliensis* Neiva, 1911 is the most important Chagas disease vector. In this study, we analyzed microevolutionary and demographic aspects of *T. brasiliensis* populations at the ecotypic, micro and macro-geographic scales by combining morphometrics and molecular results. Additionally, we aimed to address the resolution of both markers for delimiting populations in distinct scales.

**Methods:**

We sampled populations of *T. brasiliensis* from distinct ecotypic and geographic sites in the states Rio Grande do Norte (RN) and Paraíba (PB). The geometric morphometry was carried out with 13 landmarks on the right wings (*n* = 698) and the genetic structure was assessed by sequencing a region of cytochrome *b* mitochondrial gene (*n* = 221). Mahalanobis distance (MD) and coefficient of molecular differentiation (Φ_ST_) were calculated among all pairs of populations. The results of comparisons generated MD and Φ_ST_ dendrograms, and graphics of canonical variate analysis (CVA).

**Results:**

Little structure was observed for both markers for macro-geographic scales. Mantel tests comparing geographic, morphometric and genetic matrices showed low correlation (all *R*^2^ < 0.35). The factorial graphics built with the CVA evidenced population delimitation for the morphometric data at micro-geographic scales.

**Conclusions:**

We believe that *T. brasiliensis* carries in its genotype a source of information to allow the phenotypical plasticity across its whole distribution for shaping populations, which may have caused a lack of population delimitation for CVAs in morphometric analysis for macro-geographic scale analysis. On the other hand, the pattern of morphometric results in micro-geographic scales showed well-defined groups, highlighting the potential of this tool to inferences on the source for infestation.
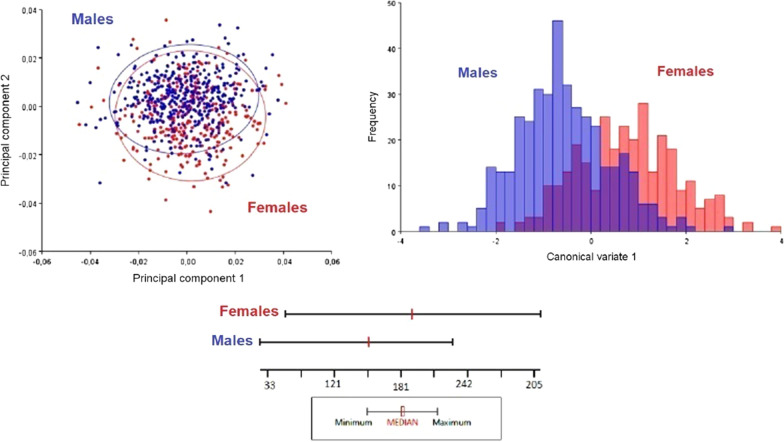

## Background

Chagas disease is among the most important neglected diseases in Latin American countries. The transmission classically occurs *via* vector insects of the subfamily Triatominae (Hemiptera: Heteroptera: Reduviidae). These insect vectors may transmit the disease when they are infected with the parasite *Trypanosoma cruzi* (Chagas, 1909) (Kinetoplastida: Trypanosomatidae), the etiological agent of the illness. Although the disease’s incidence has declined in recent decades due to intense programs to combat the main domiciliated vectors, it is estimated that six to eight million people are still infected worldwide [1].

More than 65 species of triatomines have been recorded in Brazil, but few exhibit synanthropic behaviors, such as *Triatoma infestans*, *Panstrongylus megistus* (Burmeister, 1835), *T. brasiliensis* Neiva, 1911, *T. pseudomaculata* Corrêa & Espínola, 1964 and *T. sordida* (Stål, 1859) [2]. The Brazilian Northeast, one of the poorest and underdeveloped regions of the country, raises concern in the context of Chagas disease transmission because it is an area of infestation by *T. brasiliensis* and *T. pseudomaculata*, native triatomines of difficult control and widespread in the semiarid Northeast. Whereas *T. pseudomaculata* is more associated with birds [3] (refractory to *T. cruzi* infection), *T. brasiliensis* is associated with mammals, which are potential parasite reservoirs [4, 5]. Additionally, *T. brasiliensis* is the most frequently Chagas disease vector collected in human domiciles and is considered the most important triatomine species in the region [6].

The current status of the *T. brasiliensis* species complex is based on a group of monophyletic taxa that occur in rocky outcrops of the semiarid region of the Brazilian Northeast, including seven species: *T. brasiliensis*; *T. bahiensis* Sherlock & Serafim, 1967; *T. juazeirensis* Costa & Felix, 2007; *T. lenti* Sherlock & Serafim, 1967; *T. melanica* (Neiva & Lent, 1941); *T. petrocchiae* Pinto & Barreto, 1925; and *T. sherlocki* Papa, Jurberg, Carcavallo, Cerqueira & Barata, 2002; in which *T. brasiliensis* is divided into two subspecies: *T. b. brasiliensis* Neiva, 1911 and *T. b. macromelasoma* Galvão, 1956 [7]. Lent & Wygodzinsky [8] observed that across its distribution, *T. brasiliensis* (*s.l*.) exhibited numerous chromatic forms and emphasized it should be better investigated. This led Costa and colleagues to conduct a set of investigations based on biology, ecology, phylogeny, experimental crossings [9–13] among others. The Brasiliensis subcomplex was proposed by Schofield & Galvão [14] based on the ecological data, geographical distribution and morphology, which included *T. melanocephala* Neiva & Pinto, 1923 and *T. vitticeps* (Stal, 1859) (as uncertain). The chromatic variation combined with genetic information resulted in raising *T. juazeirensi*s [15] and *T. melanica* [16] to the specific taxonomic status. One variation presenting intermediate forms was considered subspecies (*T. b. macromelasoma*), also because little genetic distance was observed with *T. b. brasiliensis* [17]. Later, *T. sherlocki* was included in this species complex by using the information on the variation in cytochrome *b* (*cytb*) and *16S* mitochondrial genes [18], which was confirmed through cytogenetic analyzes and experimental crosses [19, 20]. Other species were included in this group or revalidated, such as *T. lenti* and *T. bahiensis* [21]. Finally, *T. petrocchiae* was also considered a member of this monophyletic group, in a study combining geometric morphometrics and information on multiple mitochondrial genes (*12S* + *16S* + *cox*1 + *cytb*) [22]. The non-monophyly of the Brasiliensis subcomplex also proposed the exclusion of *T. melanocephala* and *T. vitticeps* [22], which was then confirmed through cytogenetic analyzes [23, 24]. Recently, a key was released to identify each member of this species complex [7]. For simplicity, we hereafter refer to the taxon used in this study (*T. b. brasiliensis*) as *T. brasiliensis*.

For the *T. brasiliensis* species complex, molecular studies have been mainly based on the variation of mitochondrial DNA genes [5, 17, 25] and, geometric morphometrics has already been applied for the species [26]. Molecular and morphometric markers have been combined [22, 27], but not under the focus of gamma systematics (intra-specifically). In this study, we analyzed microevolutionary and demographic aspects of *T. brasiliensis* populations at the ecotypic, micro and macro-geographical scales by combining morphometrics and molecular approaches.

## Methods

### Insects

**Fig. 1:**
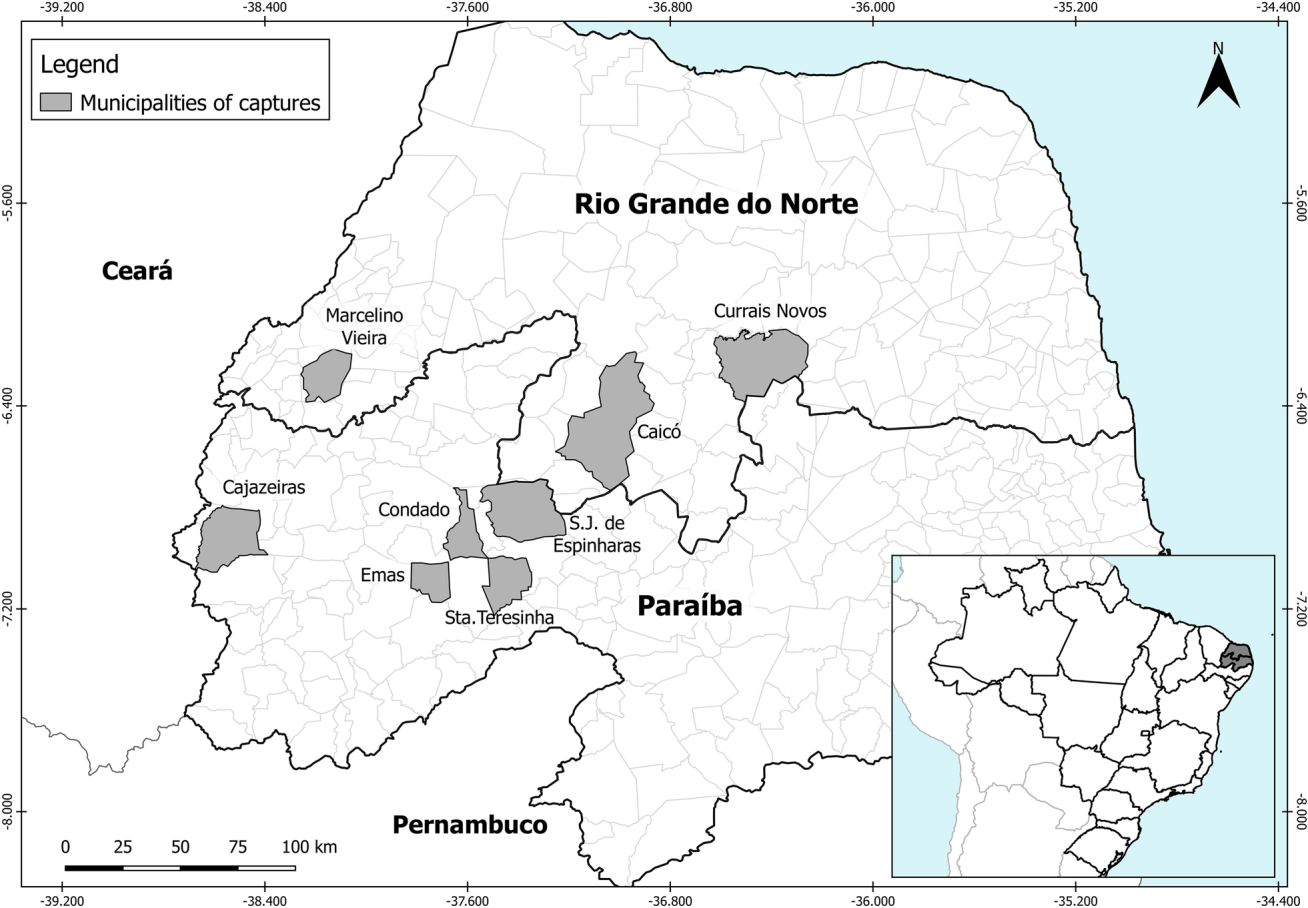
Localities (in gray) where the collections were made in the states of Rio Grande do Norte (RN) and Paraíba (PB), Brazil: Caicó-RN (CC), Condado-PB (CD), Currais Novos-RN (CN), Cajazeiras-PB (CZ), Emas-PB (EM), Marcelino Vieira-RN (MV), Santa Teresinha-PB (ST), São José de Espinharas-PB (SJ) and São Mamede-PB (SM)

Triatomine captures were conducted in the State of Rio Grande do Norte (RN), in the municipalities of Caicó, Currais Novos and Marcelino Vieira, whereas in the State of Paraíba (PB), insects were collected in the municipalities of Condado, Cajazeiras, Santa Teresinha, São José de Espinharas, São Mamede and Emas (Fig. 1). The sampling covered a range of 240 km (East-West) and 95 km (North-South) (− 6°58′48.0″ to − 6°08′49.2″ latitude and − 38°35′27.6″ to − 36°29′09.6″ longitude). The sampled spots were within the biogeographical zone known as Caatinga, a mosaic of xerophytic, deciduous, semiarid thorn scrub and forest [28]. All captures were conducted manually with the aid of tweezers. We defined three distinct ecotopes: (i) the sylvatic ecotopes (primary) were represented by rocky outcrops; (ii) the domestic ecotopes were considered as the indoor spaces where humans sleep or work; and (iii) the peridomestic ecotopes were defined as the spaces surrounding domiciles, where domesticated animals sleep or are raised. In peridomiciles, most of the triatomines were captured in stone/woodpiles, chicken coops and goat and pig corrals. Captured specimens were identified according to taxonomic keys [7, 8, 24]. The insects were individually labeled and stored in 1.5 ml microtubes with 100% ethanol. The wings were kept dry in another tube with the same label for the ones in ethanol. In the laboratory, all tubes were stored at < 5 °C.

### Population definition

We based the population defined as the set of insects collected at the same site and capture spot. Each insect/wing was cataloged at the population level, receiving identification acronyms, namely: (i) the first two letters refer to the locality: Caicó-RN (CC); Currais Novos-RN (CN); Marcelino Vieira-RN (MV); Condado-PB (CD); Cajazeiras-PB (CZ); Santa Teresinha-PB (ST); São José de Espinharas-PB (SJ); São Mamede-PB (SM); and Emas-PB (EM) (Fig. 1); (ii) geographical coordinate (two/three numbers); (iii) ecotope: wild (S); peridomestic (P); and domestic (D); and (iv) sex: male (M); and female (F). Morphometrics and molecular analyses were carried out for the same populations.

### Morphometric analysis

**Fig. 2:**
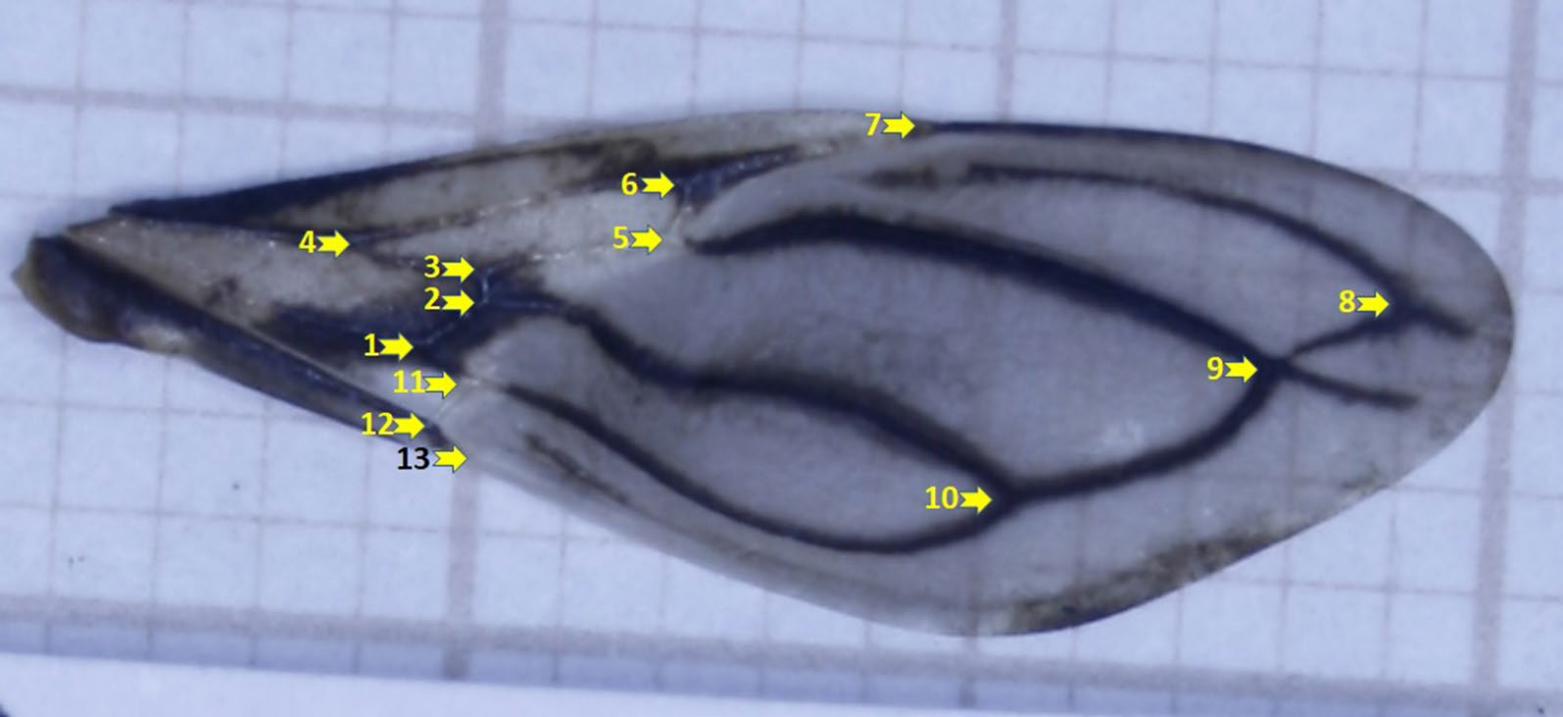
Landmarks used for the analyses. The illustration is a right (anterior) wing of a male *Triatoma brasiliensis* from the wild population of Caicó (RN), Brazil

For this study, the anterior wing of 5–30 individuals per population was used. Considering the epidemiological importance, we did not exclude populations with lower numbers, as for the bug collected inside domiciles in Condado-PB. Photographic records of right wings were made using a stereomicroscope Zeiss™ Discovery V.12 (Göttingen, Germany) with the image capture system AxioCam 5.0™ and ZEN™ software version 2.0 (Carl Zeiss, SteREO Discovery). Thirteen anatomical landmarks (Fig. 2) were used *via* TPS dig 2.17 [29]. To reduce the user effect, the same operator marked all landmarks. Additionally, the wing images of each sex were mixed and picked up randomly before capturing the landmarks.

The shape variables were obtained by using the generalized Procrustes superposition algorithm and the subsequent projection of the Procrustes residues were defined in an Euclidean space. Both non-uniform and uniform components were used as shape variables [30]. These two components describe the differences in deviations from an average reference point configuration [31]. The centroid size of the isometric estimator (CS) derived from the analysis based on anatomical landmarks was used as a measure of the overall size [29–33].

For shape measurements, Mahalanobis distances (MD) between all pairs of populations were calculated and their significance was assessed using a non-parametric test based on permutations (10,000 simulations), which were illustrated in dendrograms. The choice for MD instead of Procrustes distances (PD) was based on some studies that say that PD produces estimates that are statistically inconsistent if the variation is not isotropic [34–36]. Additionally, pilot runs showed little difference between PD and MD. The percentage of phenotypic similarity between pairs of populations was calculated using a cross-validation test, which evidences the percentages of individuals that were correctly assigned to their populations. The relationship between centroid size (CS) and discrimination of shapes between groups (allometry) was estimated through a multivariate regression between the coordinates of Procrustes (dependent variables) and CS (independent variable). This analysis was performed for each module and among populations/population groups. These regressions show associations in all comparisons *via* random permutations. The degree of differentiation between individuals was illustrated through graphics of principal components analysis (PCA) and canonical variate analysis (CVA), which were assessed by the combination of variations in the 13 anatomical coordinates of each individual. All of these statistics were performed using the MorphoJ software [33]. *P*-values were considered significant at *P* < 0.05.

### Molecular analysis

The total DNA of 3 legs was extracted using the Qiagen Blood and Tissue kit (Valencia, CA, USA), following the manufacturer’s specifications. The mitochondrial cytochrome *b* (*cytb*) gene was amplified by the polymerase chain reaction (PCR), using the primer pair CYTB7432F, and CYTBR and amplification conditions set by the authors who designed the primers [37, 38]. Both strands (forward and reverse) were sequenced by using the purifications and sequencing reactions defined in [37, 38]. Forward and reverse sequences were assembled into contigs for editing [39]. Edited sequences were compared to those in the GenBank database using the BLAST [40] program to check the specific status. Sequences were trimmed to 447 bp so as they were of equal length. A phylogram was built with the resulting *cytb* sequences *via* Bayesian inference with unique haplotypes. The matrix was run in BEAST 1.8.0 [41] with 10 million generations and sampling every 1000 simulations. A strict molecular clock model (with a default rate of 1.0) and the constant-size coalescent tree prior were applied for this analysis. Tracer 1.7 [42] was used to check parameter convergence with a stationary state, using a minimum threshold for ESS (effective sample size) value of 200. TreeAnnotator (available in the BEAST package) was used to summarize all the generated trees with the highest credibility, being visualized and edited using FigTree 1.4.4 (http://tree.bio.ed.ac.uk/software/figtree/). The evolutionary model was chosen according to the Bayesian information criterion (BIC) in MrModeltest [43]. Estimates of evolutionary divergence between sequences were based on Kimura 2-parameter model (K2P) [44] by using MEGA-X software [45].

For population analysis, summary statistics of genetic diversity of *T. brasiliensis* were calculated for insects from each population. The null hypothesis of neutrality was tested using Tajima’s *D* statistic [46] and Fu’s Fs [47]. The following population genetic summary statistics were calculated; number of haplotypes (H), haplotype diversity (Hd) and nucleotide diversity (π). A hierarchical analysis of molecular variance (AMOVA) [48] was performed to access the population’s genetic structure. DnaSP v6 software [49] was used to generate the files to run the analyses described above in Arlequin 3.5 [50] software. The significance of molecular statistics (Haplotype diversity, Pi [π], Fu’s, Tajima’s D) [46] was tested using 1,000 permutations, being considered significant those at *P*-values < 0.05.

## Results

### Morphometrics

Overall, 733 wings were digitized and had the anatomic coordinates marked. Forty-one samples were considered outliers and excluded. The 692 samples were distributed in 43 different populations. The first two PCA axes explained 36% of the variation for the total sample. The first comparison was made between the sexes, with 298 females and 394 males. The first plane of the PCA (Additional file 1: Figure S1a) showed a higher concentration of females on the negative pole of component 2. We calculated the Mahalanobis distance between sexes (MD = 1.343), which was statistically significant (*P* < 0.0001). The cross-validation test wrongly classified 27–28% of the individuals according to sex (Additional file 1: Figure S1b). The box-plot (Additional file 1: Figure S1c) for the centroid size showed that the females were larger than males (*P* < 0.0001), as expected. Thus, we assumed that sexual dimorphism may have influenced differentiation and dealt with sexes separately. We excluded populations with low number of samples (*n* < 5). Therefore, the resulting dataset included 349 samples from 24 populations for males (Additional file 2: Table S1) and 284 specimens from 25 populations for females (Additional file 2: Table S2).

**Fig. 3:**
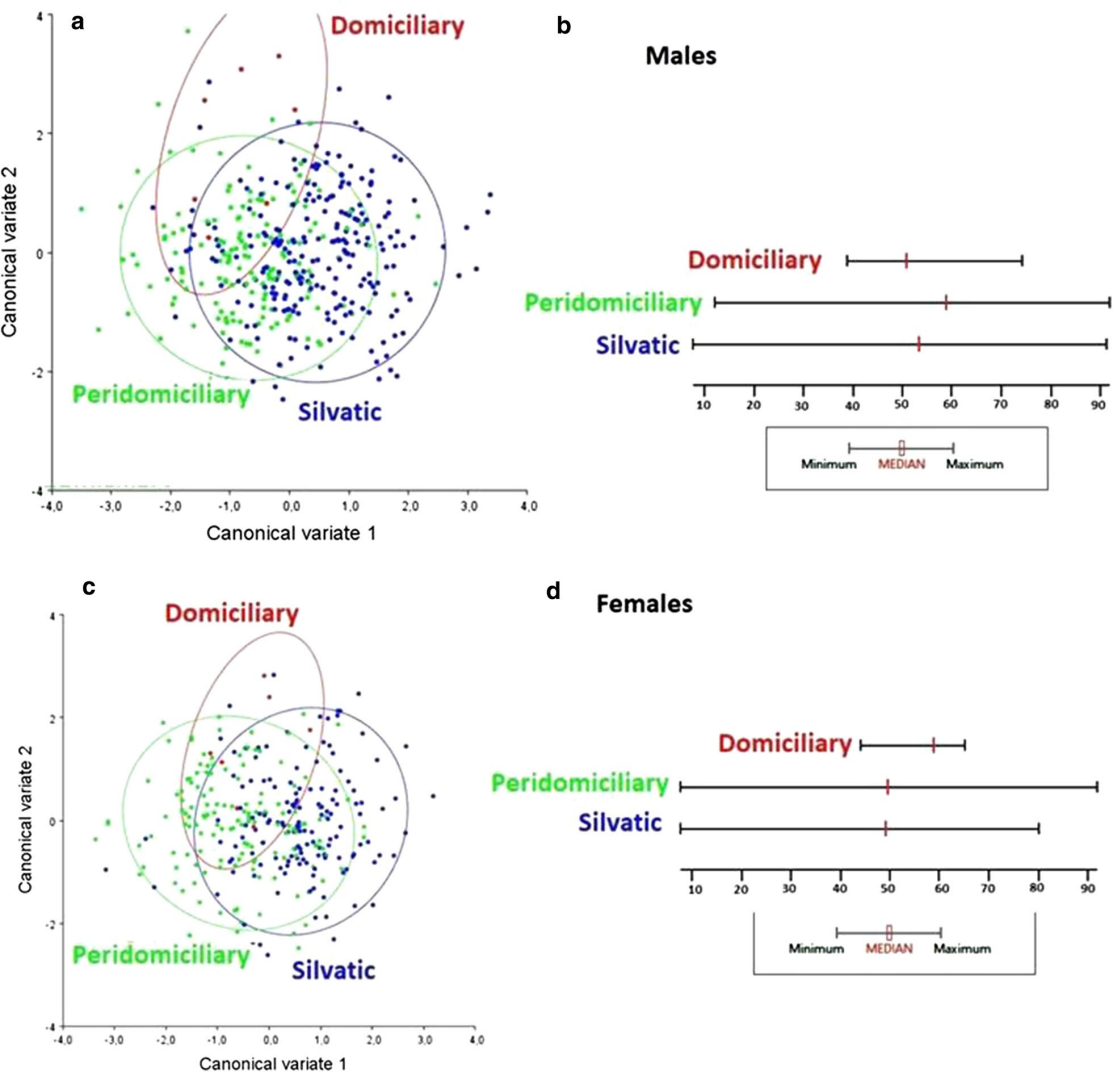
Canonical variance for groups of *Triatoma brasiliensis* populations collected in distinct ecotopes are illustrated in **a** (males) and **c** (females). The sizes are based on the centroid for overall males (**b**) and females (**d**)

Figure 3 shows that insects were sorted according to the ecotope; peridomestic insects tended to be at the left pole of CV1 whereas sylvatic at the right pole of CV2, which exhibited a statistically significant MD (all > 1.21; *P* < 00001) irrespective of sex. Both sexes were larger in peridomestic insects (Fig. 3a–d), but domestic insects had smaller males (Fig. 3b) and larger females (Fig. 3d). However, domestic populations were represented by only 14 insects, divided into 5 populations.

When samples were grouped according to the municipality of collection (irrespective of collection site and ecotope), it was not possible to observe any grouping delimitation in the CVA plot in relation to sex (Additional file 3: Figure S2), even if the samples were sorted according to the ecotope within each municipality (data not shown, because the pattern remains the same).

**Fig. 4:**
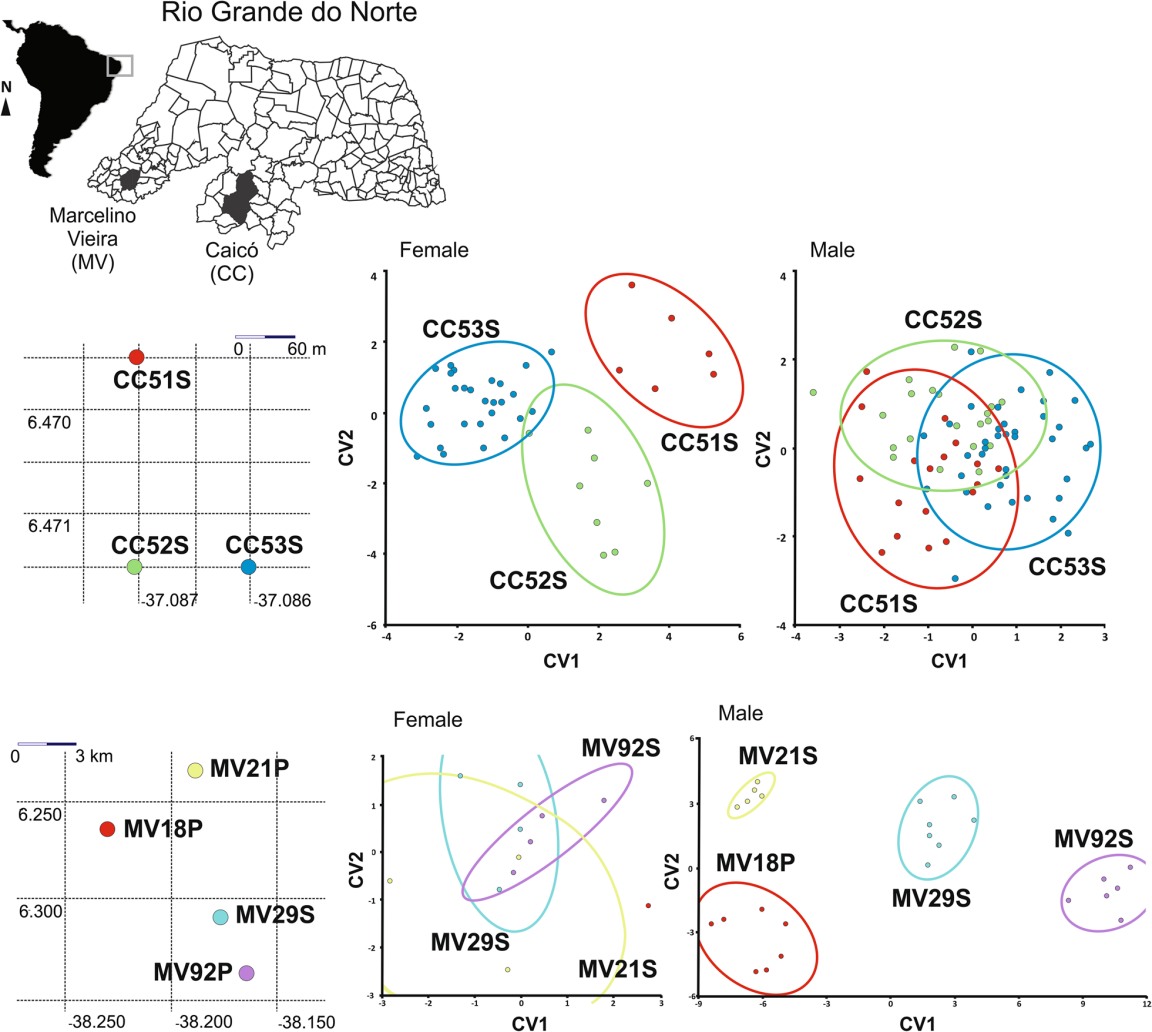
Left: The locations where *Triatoma brasiliensis* populations were collected in Caicó and Marcelino Vieira in the State of Rio Grande do Norte are on the left side. Right: plots based on the first and second canonical variate analysis for both sexes

In a CVA run within each municipality, almost all groups were delimited. The clear exception was for both sexes for populations from Currais Novos (Additional file 4: Figure S3). In Caicó-RN, females from the three locations of capture showed morphometric delimitation, whereas the males did not exhibit such structuring. The opposite was observed for the populations from Marcelino Vieira (Fig. 4).

**Fig. 5:**
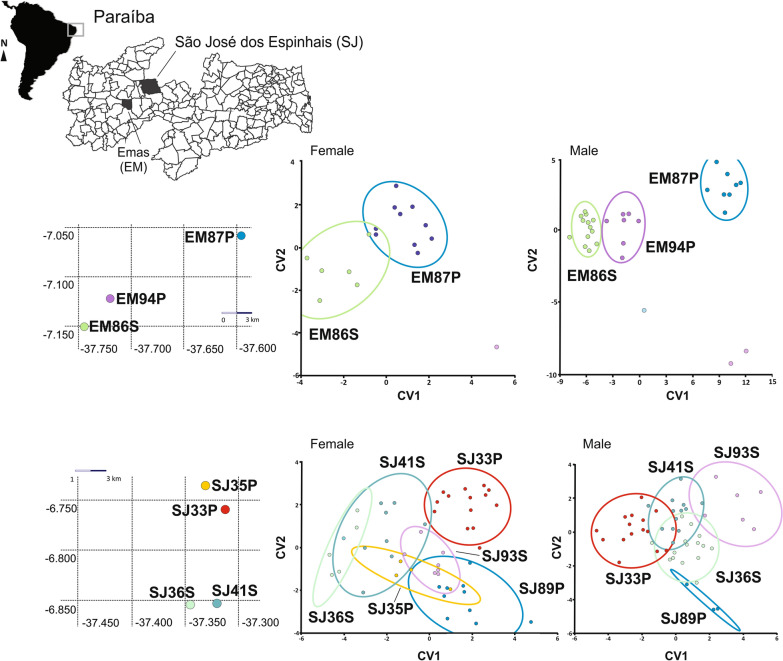
Left: The localities where *Triatoma brasiliensis* populations were collected in Emas and São José dos Espinhais in the State of Paraíba. Right: plots based on the first and second canonical variate analysis for both sexes

For Emas-PB, all populations were delimited. However, for females, we did not have enough samples to compose the third population (EM94P). Populations from São José dos Espinhais-PB also showed some well-delimited groups for both sexes, even though there was some overlap (Fig. 5).

**Fig. 6:**
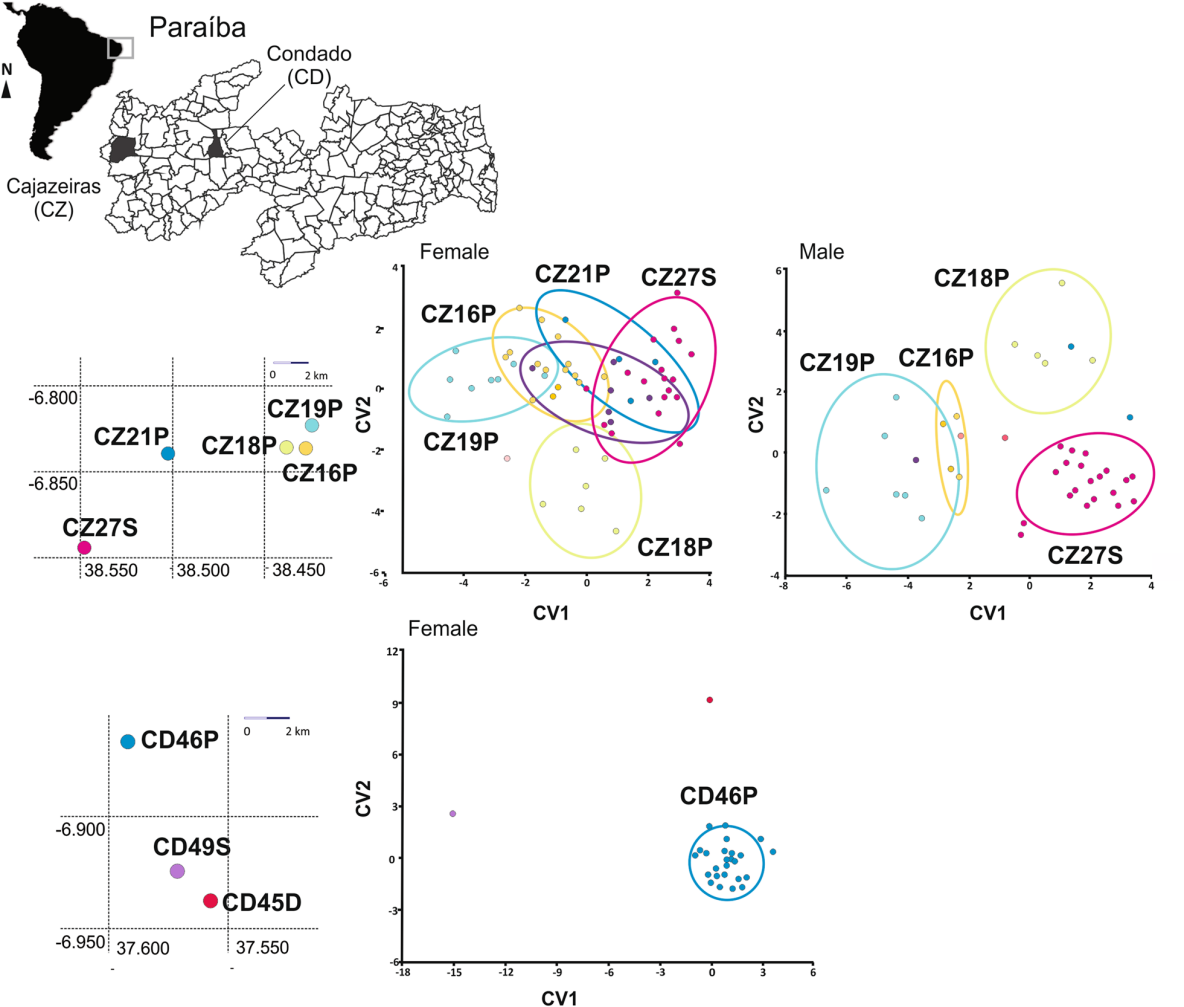
Left: The localities where *Triatoma brasiliensis* populations were collected in Cajazeiras and Condado in the State of Paraíba. Right: plots based on the first and second canonical variate analysis for both sexes. We did not have enough males for all populations from Condado

For Cajazeiras, clear delimitation was observed for males. For females of Cajazeiras, the peridomestic populations CZ18P and CZ19P were delimitated and there was an overlap for the remaining, that was composed of populations from distinct ecotopes. For Condado municipality, we only had a sufficient number for morphometric analysis for females but we observed that this peridomestic population (CD46D) was delimited in a group separated from others, which were isolated in distinct areas of the graph (Fig. 6).

### Molecular variation in *cytb* gene

Sequences were generated from 221 individuals, distributed in 30 populations. The number of individuals sequenced per population varied from 3 to 10, but two populations were represented by only one sequence each. Overall, 57 segregating sites, 52 haplotypes (GenBank: MT720956-MT721007, Additional file 2: Table S4) and a haplotype diversity of 0.92 were observed. Nucleotide diversity was 0.00601. The Tajima’s *D* and Fu’s Fs tests indicated that, in general, DNA sequences are evolving randomly (“neutral”). The exceptions were for the wild populations of Caicó (CC51S; Tajima’s *D*), the peridomestic area of Currais Novos (CC69P and CN86P; for Fu’s Fs and Tajima’s *D*). The highest haplotypic diversity was observed for the populations CC51S (0.9556 ± 0.0594). The π value for SJ41S population was also the highest (0.003853 ± 0.002802), followed by MV29P (0.002486 ± 0.001997) and MV216S (0.001491 ± 0.001424), being below 0.001 for the remaining populations. The number of samples, haplotypes and remaining molecular statistics are provided in Additional file 2: Table S3. Additional file 2: Table S4 shows the estimates of evolutionary divergence between sequences based on K2P. All values were low (< 0.01), indicating that we were dealing with *T. brasiliensis*. The phylogenetic reconstruction by using unique haplotypes based on Bayesian inference revealed that posterior probabilities were low in general. For cases where these were higher than 80%, sequences had K2P lower than 0.009, confirming we were dealing with haplotypes with low variation. The molecular evolution model was HKY + G based on BIC (Additional file 5: Figure S4).

**Fig. 7:**
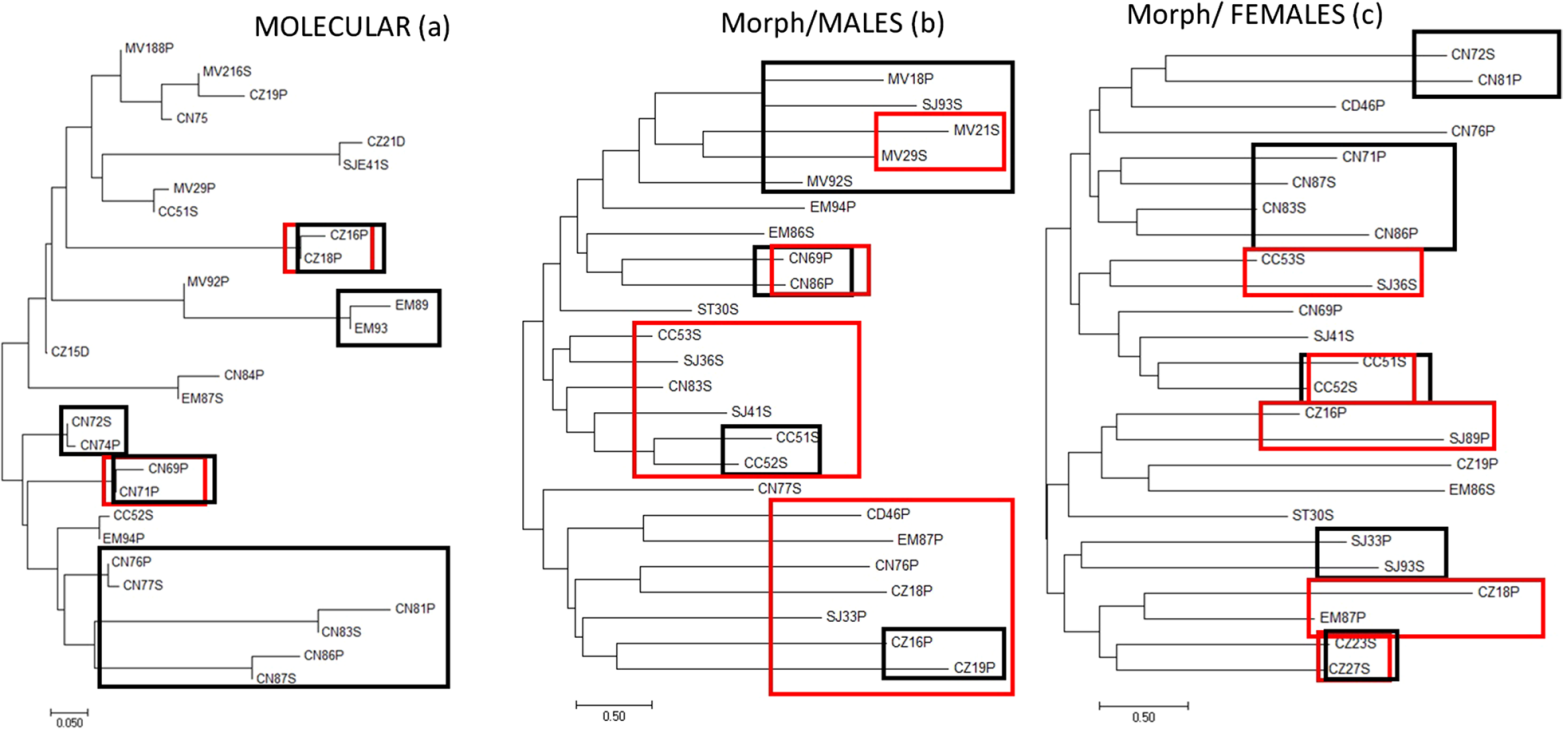
Trees derived from pairwise molecular differentiation coefficient (**a**) and morphometric analyses Mahalanobis distances for males (**b**) and females (**c**) of *Triatoma brasiliensis* populations. Details for each population of *T. brasiliensis* are provided in Additional file 2. Black rectangles refer to geographical related populations and the red rectangles indicate populations ecotypic-related

The Φ_ST_ tree is illustrated in Fig. 7. The values of Φ_ST_ are given in Additional file 2: Table S5. The tree built with genotypic data showed the geographical force of the distribution of genetic variation. For example, the majority of Currais Novos (CN#) populations are associated with the same branch (Fig. 7a). Morphometric results exhibited geographical groupings as for males of Marcelino Vieira (MV#) (Fig. 7b), but also ectopic, indicated by red rectangles. Most of the morphometric population comparisons were statistically significant (Additional file 2: Table S1 for males and Additional file 2: Table S2 for females). We highlight the discrepancies of the trees based on genetic and morphometric data: the tree based on genetic data exhibited more consistent geographical groupings whereas the trees based on morphometrics exhibited groupings for both scales (geographical and ecotypic) (Fig 7b, c).

Analysis of molecular variance (AMOVA) (Table 1) demonstrated that when populations are grouped according to the municipality, 18% of the variation is among municipalities and 36% among populations within municipalities. However, variation among groups of municipalities was significant (*P* = 0.001). Following the hypothesis that the genetic structure may be driven by ecotypic force (predominant migration between the same ecotopes), a second test was performed by nesting groups of populations defined by their ecotopes within each municipality. For this kind of nesting, the percentage of variation between groups was even lower (10%) and the variation within groups was higher (41%). For both kinds of nesting populations, most of the variation was observed within populations (46–49%).

**Table 1:** Analysis of molecular variance (AMOVA). Percentage of variation explained at each spatial/ecotopic level and fixation indexes for grouping according to municipalities or for *Triatoma brasiliensis* ecotypic populations within municipalities in Rio Grande do Norte and Paraíba, Brazil

	*df*	Variance components	Percentage of variation	Fixation indices	*P*-value
Variation between municipalities	3	0.29 Va	18	0.17 F_CT_	0.0010
Among populations within municipalities	11	0.58 Vb	36	0.76 F_SC_	< 0.0001
Within populations	115	0.76 Vc	46	0.54 F_ST_	< 0.0001
Between groups of ecotypic populations in each municipality	4	0.16 Va	10	0.10 F_CT_	0.050
Among populations within groups	10	0.63 Vb	41	0.46 F_SC_	< 0.0001
Within populations	115	0.76 Vc	49	0.51327 F_ST_	< 0.0001

### Correlation between matrices

All Mantel tests comparing the matrices showed a significant correlation (*P* < 0.05) but in general, correlations were low. The highest correlation occurred between the MD between males and females (*R*^2^ = 0.74), as expected. The remaining correlations exhibited low values, such as between the Φ_ST/_MD and the geographical distance (*R*^2^ < 0.36). For both sexes, the correlations between MD and Φ_ST_ were also low (*R*^2^ = 0.13–0.15).

## Discussion

The differences between the distributional patterns of morphometric and genetic variation have been extensively explored for insect vectors [51–53]. For the *T. brasiliensis* complex, interspecific molecular and morphometric variations have also been studied [22]. Population variations based on morphology can be influenced by ecotypic and environmental variations, but they are also influenced by the individual’s genotypic information [54]. Therefore, geometric morphometrics approaches are also used to recognize microevolutionary processes [32]. The first study in this sense for *T. brasiliensis* used classical morphometry [55]. The second study was conducted at a microgeographical scale perspective and had a restricted sampling number (*n* = 97), but it was a pioneer study by using geometric morphometrics for assessing the variation among ecotypic populations [26]. Our study brings up the first effort by using a large sampling of *T. brasiliensis* (initial *n* of 733 insects) on a wider geographical scale ranging from 95 km (North-South) to 240 km (East-West). Additionally, the morphometric information is here associated with genotypic variations. Some authors [56, 57] concluded that morphometrics combined with genetic information can improve the understanding of the re-infestation process and contribute to vector control strategies when the approach is used in the microgeographical scale. In this study, molecular variation was used as a tool for monitoring and control, as the chosen marker (variation in the mitochondrial gene *cytb*) does not suffer the influence of the environmental variation [58]. Thus, this study is also a pioneer in contrasting robustally the ecotypic effect on morphometric variation for Brazilian Chagas disease vectors.

Considering all the samples grouped according to the ecotopes and for each sex, we observed a relative ecotypic grouping in the PCA. Similarly, in Ceará, Batista et al. [26] found significant differences between *T. brasiliensis* collected in different ecotopes. The peridomestic individuals were significantly larger than the sylvatic individuals. Costa et al. [13] mentioned that wild populations of *T. brasiliensis* are usually found hungry in the field, which may explain the difference in size. We suggest that domestic insects did not have numbers enough for inferring the size. Geometric morphometrics effectively minimizes the size factor, but it does not eliminate it totally [32]. The ecotopes seem to influence the morphometric variation, which may represent the bias of geometric morphometry for inferences on genetic structuring. However, as discussed below, some peridomestic populations were more morphometrically associated with some sylvatic bug populations than other peridomestic populations, when the observation is carried out on a microgeographical scale.

In the dendrograms derived from the Mahalanobis distances (MD), it was possible to observe that some groups were formed by nearby populations, indicating geographical structure. The District of Patos is composed of the municipalities of Emas, São José de Espinharas, Santa Teresinha, Condado, all in the State of Paraíba with related bug populations. However, the Mantel test showed a weak correlation between the morphometric and geographical distance matrices, which may indicate that forces, other than geographical, maybe also driving the morphometric variation. For *T. infestans* in Argentina, the resolution of geometric morphometry to detect differences in macrogeographical scales was more evident in comparisons of individuals separated by municipality [56]. However, the authors did not apply any matrix correlation tests (Mantel tests). Additionally, these authors did not include insects from wild populations.

Principal components analysis (PCA) is the most popular method to characterize shape variation [33]. However, when the data involve subgroups, the PCA should be used as a method for ordering large groups, with the canonical variates analysis (CVA) being the most appropriate for the subgroups. The factorial maps constructed with the CVAs in populations defined by collection point within each municipality (on a microgeographical scale) were the only maps to delimit the populations. Pilot tests with PCAs could not delimit populations even for macroeographical scales.

For both sexes, no structure was observed for the populations from Currais Novos; and for Caicó there was grouping delimitation only for females in CVA graphs. The lack of correlation between the CVA graphs in regard to sexes for Caicó may indicate a different migration pattern. However, for overall results, the Mantel test showed 73% correlation between males and females for MDs. For *T. dimidiata* (Latreille, 1811), Stevens et al. [59] found no significant differences in gene flow between the sexes using microsatellite markers. For *T. juazeirensis*, a member of the *T. brasiliensis* complex, Carbajal de la Fuente et al. [60] found more males than females in light traps, which capture flying insects (dispersants). Lilioso et al. [61] captured *T. brasiliensis via* active search, and they found more than twice males than females in sylvatic environments. This may indicate the existence of distinct patterns of migration between sexes.

We found low variation among *T. brasiliensis* sequences (all K2P were < 0.01). This value is expected intraspecifically since the values reported between the subspecies *T. b. brasiliensis* and *T. b. macromelasoma* were higher (K2P = 0.03) [17]. Therefore, we assumed we were dealing with closely related entities. For mosquitoes, Lorenz et al. [62] presented interesting clues about selective patterns observed by molecular markers, which were corroborated by morphometric markers. However, the neutrality tests here applied pointed to a random (“neutral”) evolution for our sampling. In other words, we did not evidence directional or balancing selection, as well as expansion or demographic contraction. Therefore, we could not provide inferences on molecular and morphometric results in this sense. Indeed, the neutral evolution for the molecular marker chosen is in agreement with the results of Almeida et al. [25] which used the same gene fragment in Caicó-RN [5] and also in São José da Lagoa Tapada-PB [5].

The hierarchical molecular variance analysis (AMOVA) was conducted to evaluate the role of geographical and ecotypic forces in the genetic structure of *T. brasiliensis*. In this case, it is known that the ecotope does not influence the genotype, but for some triatomine vectors, gene flow occurs predominantly between ecotopes, as observed for *T. infestans* in the Andean valleys [63]. Here, neither the geographical force nor the ecotypic one explained the distribution of molecular variation, based on the observation that the greater percentage of variation was detected within groups and within geographical and ecotypic populations. Additionally, Mantel tests showed a low correlation between geographical and genetic variation. These results corroborate Almeida et al. [5, 25], although these authors have noticed some degree of influence of ecotypic force. However, at this point, the morphometric and molecular results were in agreement. What is noteworthy, is that the molecular and morphometric markers were in agreement to indicate an association between a sylvatic and a peridomestic population of Marcelino Vieira (MV188P-MV216S; Figs. 4, 6a). It is important to note that the population MV188P was collected at the site where a Chagas disease outbreak occurred [64].

Both molecular and morphometric markers can be applied to understand macroevolutionary and microevolutionary processes in vector insects [65]. Concerning microevolutionary processes, in general, the dendrogram constructed with the Φ_ST_ matrices revealed that the geographical forces play a role in the distribution of genetic variation. However, most populations of Currais Novos were in the same cluster for morphometrics of males from Marcelino Veira. This was observed for some populations of Cajazeiras and Emas, although AMOVA has not shown statistical significance for geographical structuring. On the other hand, this corroborates the results of morphometry, in which Mantel tests did not evidence correlation between matrices of geographical and Mahalanobis distances.

The comparison of the results of the molecular and morphometric tools showed low conformity by Mantel tests. But in all dendrograms, the populations of Cajazeiras and Currais Novos trend to be grouped in the same branch. This indicates that in some cases the markers are in agreement. This conformity has already been tested by other authors and for other species, such as *T. dimidiata* (Latreille, 1811) in the Yucatan Peninsula in Mexico [66] and *T. infestans* in Argentina [56]. For *T. dimidiata* the authors did not find remarkable conformity between markers [66], which may be explained by the distinct resolution of molecular and morphometric markers [67].

The strategy of observing variations within a smaller geographical scale aimed to seek clues about the processes of (peri) household infestation. To our knowledge, this is the first attempt in the literature to test geometric morphometrics for this purpose focusing on vectors of Chagas disease in Brazil. Schachter-Broide et al. [68] applied the approach to this inference for *T. infestans* in Argentina, concluding that geometric morphometrics represents a useful tool. However, the populations analyzed by these authors all came from artificial ecotopes. We must emphasize that our results suggest that the ecotope seems to have an important effect on phenotypic variation. Therefore, the morphometric associations between populations of different ecotopes on the microgeographical scale can present important clues to detect infestation processes when analyzed within the same municipality. In this sense, geometric morphometrics was more sensitive to delimit populations on a microgeographical scale than on a macrogeographical scale, which was evidenced in the factorial maps. For example, in the maps illustrating the analysis of canonical variables for both males and females, the peridomestic populations of Cajazeiras and São José de Espinharas showed associations with sylvatic populations. These findings corroborate the idea that this tool can be used to detect infestation foci [32]. However, caution is needed for this conclusion. We recommend the combination of morphometry with molecular markers of higher resolution, such as single-nucleotide polymorphisms (SNPs) [69] to validate morphometric results.

The genome of *T. brasiliensis* may carry information that allows equal morphological plasticity throughout the entire collection range which prevents structuring on the macrogeographical scale. However, demographic events, such as bottlenecks or founder effects, might be acting to maintain homogeneity in groups of insects collected at the same point, as recently observed for Brazilian populations of *T. sordida* [70]. Retention of the ancestral character and convergences in sampled populations can also help explain the results of morphological plasticity. The retention was already verified by molecular tools: the ancestral haplotype of *cytb* is the most frequent in the entire collection range, and this phenomenon is well known for *T. brasilensis* [5, 17, 25]. Almeida et al. [25] could not delimit populations using the *cytb* gene on a microgeographical scale. The power demonstrated by geometric morphometrics to delimit populations on a microgeographical scale indicates that this tool has a higher resolution in revealing population structure than the molecular marker used here.

The ecologic niche model was used for the first evaluation of the distributional potential of members of *T. brasiliensis* in the present and also for future climate scenarios - global warming. Model projections indicated the potential for expansions of *T. brasiliensis* for 2050, highlighting the need for constant monitoring *T. brasiliensis* distribution [71].

## Conclusions

The most remarkable result obtained here was the resolution in the population delimitation on a microgeographical scale for geometric morphometrics, which was sometimes in agreement with the results of molecular variation. At this scale, populations from distinct ecotopes appeared to be more associated in some cases, which questions the role of the ecotope on the distribution of morphometric variation. This indicates that geometric morphometrics can be used to assess population structure, microevolutionary processes and to look for foci of reinfestation. The combination of molecular and morphometric markers have the potential to be used in decision-making for more rational, targeted and resource-saving control measures (as for spraying domiciles with insecticides). Additionally, the geometric morphometrics based on the venation of the wings of triatomines is a low-cost and quick-to-perform technique. Therefore, the results presented here indicate that there is potential applicability of this technique in vector control measures in the near future.

## Supplementary information

**Additional file 1: Figure S1**. **a** PCA for the morphometric variation of all *T. brasiliensis*. Red dots represent females and blue dots represent males. **b** The canonical variable using the discriminating function of the variation between the sexes. **c** Boxplots representing variations in the size of the wing centroid.

**Additional file 2: Table S1.** Statistics for the Mahalanobis distance of males. **Table S2.** Statistics for the Mahalanobis distance of females. **Table S3.** Summary statistics of genetic diversity. **Table S4.** Estimates of evolutionary divergence between cyt B fragments (447bp). **Table S5.** Φ_ST_ pairwise population comparisons for all populations.

**Additional file 3: Figure S2.** Graphics of Canonical Variance for males (**a**) and females (**b**) *T. brasiliensis* populations grouped by municipality. See Fig. 1 for the acronyms of municipalities. *Abbreviations*: CC, Caicó-RN; CD, Condado-PB; CN, Currais Novos-RN; CZ, Cajazeiras-PB; EM, Emas-PB; MV, Marcelino Vieira-RN; SJ, São José de Espinharas-PB; ST, Santa Teresinha-PB.

**Additional file 4: Figure S3.** Left: The locations where *T. brasiliensis* populations were collected in CurraisNovos, State of Rio Grande do Norte. Right: Canonical variate analysis (CVA) based on CVA1 and CVA2 for both sexes.

**Additional file 5: Figure S4.** Bayesian phylogenetic tree based on the *cytb* gene (447 bp) for *T. brasiliensis* using the molecular evolution model TN93 + I. Posterior probabilities are indicated at the nodes. Outgroup: *T. petrocchiae* (GenBank: MN177967).

## Data Availability

All data generated or analyzed during this study are included in this published article and its additional files. The newly generated sequences were deposited in the GenBank database under the accession numbers MT720956-MT721007.

## Abbreviations

MD: Mahalanobis distance
CVA: canonical variate analysis
AMOVA: analysis of molecular variance
PCA: principal components analysis
RN: Rio Grande do Norte State, Brazil
PB: Paraíba State, Brazil
CC: Caicó City, Brazil
CN: Currais Novos City, Brazil
MV: Marcelino Vieira City, Brazil
CD: Condado City, Brazil
CZ: Cajazeiras City, Brazil
ST: Santa Terezinha City, Brazil
SJ: São José dos Espinharas City, Brazil
SM: São Mamede City, Brazil
EM: Emas City, Brazil
