# Supplementary figures and images for: Drivers of molecular and morphometric variation in Triatoma brasiliensis (Hemiptera: Triatominae): the resolution of geometric morphometrics for populational structuring on a microgeographical scale
Source: Parasit Vectors. 2020 Sep 7;13:455. doi: 10.1186/s13071-020-04340-7 (PMC7487581; doi:10.1186/s13071-020-04340-7)

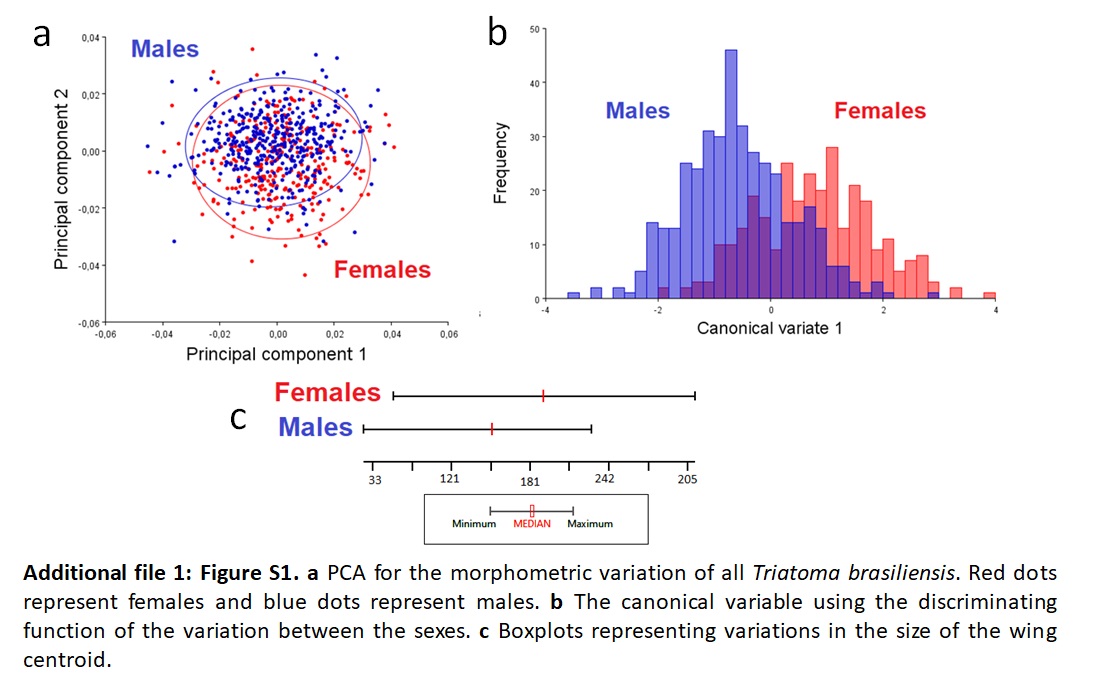

Supplement: Supplementary file 1 — Additional file 1: Figure S1. a PCA for the morphometric variation of all T. brasiliensis. Red dots represent females and blue dots represent males. b The canonical variable using the discriminating function of the variation between the sexes. c Boxplots representing variations in the size of the wing centroid. [file 13071_2020_4340_MOESM1_ESM.jpg]

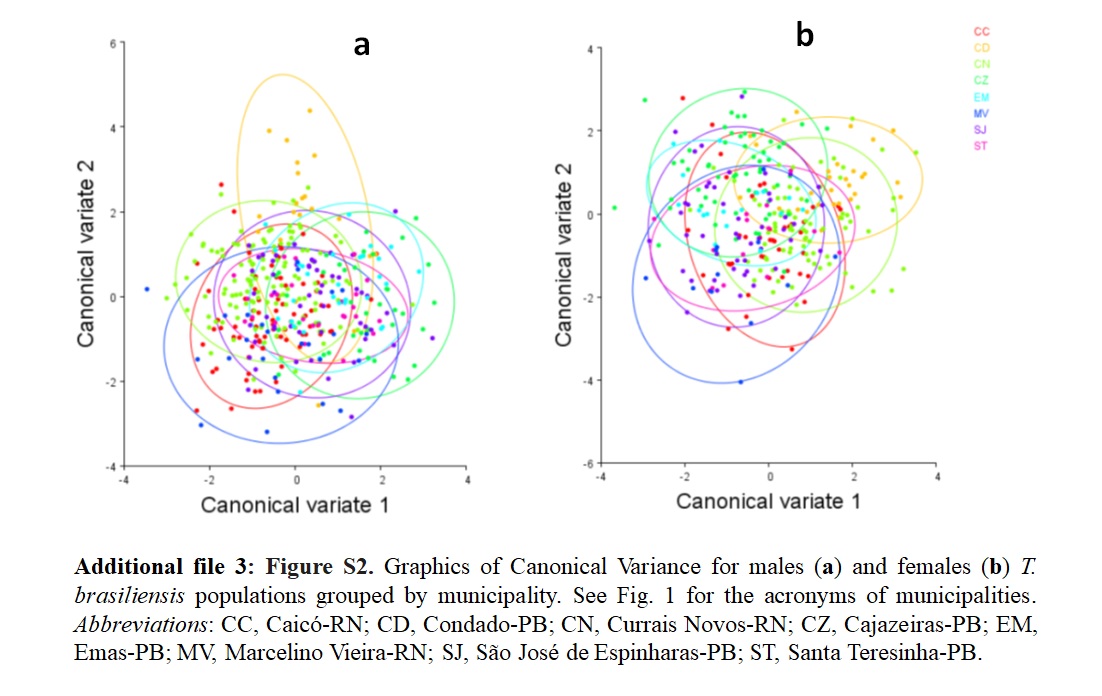

Supplement: Supplementary file 3 — Additional file 3: Figure S2. Graphics of Canonical Variance for males (a) and females (b) T. brasiliensis populations grouped by municipality. See Fig. 1 for the acronyms of municipalities. Abbreviations: CC, Caicó-RN; CD, Condado-PB; CN, Currais Novos-RN; CZ, Cajazeiras-PB; EM, Emas-PB; MV, Marcelino Vieira-RN; SJ, São José de Espinharas-PB; ST, Santa Teresinha-PB. [file 13071_2020_4340_MOESM3_ESM.jpg]

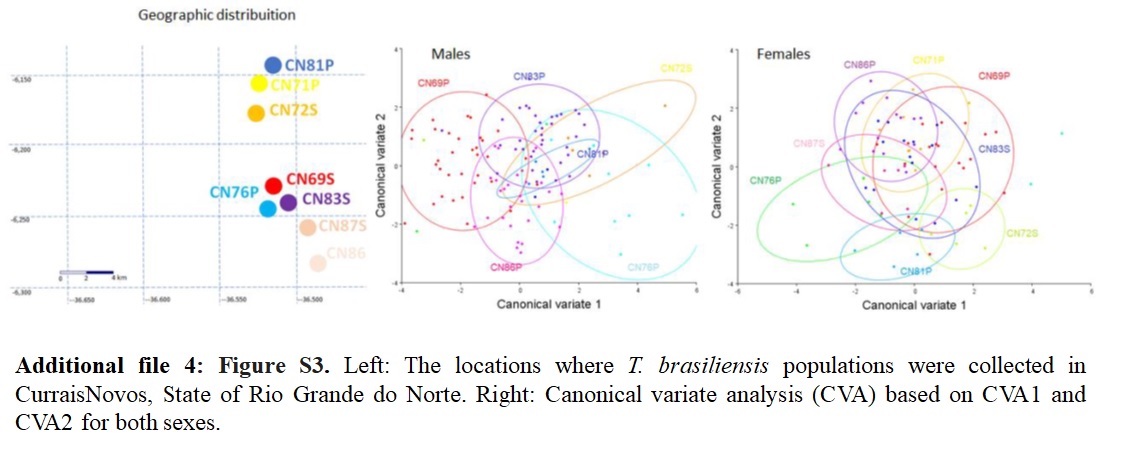

Supplement: Supplementary file 4 — Additional file 4: Figure S3. Left: The locations where T. brasiliensis populations were collected in CurraisNovos, State of Rio Grande do Norte. Right: Canonical variate analysis (CVA) based on CVA1 and CVA2 for both sexes. [file 13071_2020_4340_MOESM4_ESM.jpg]

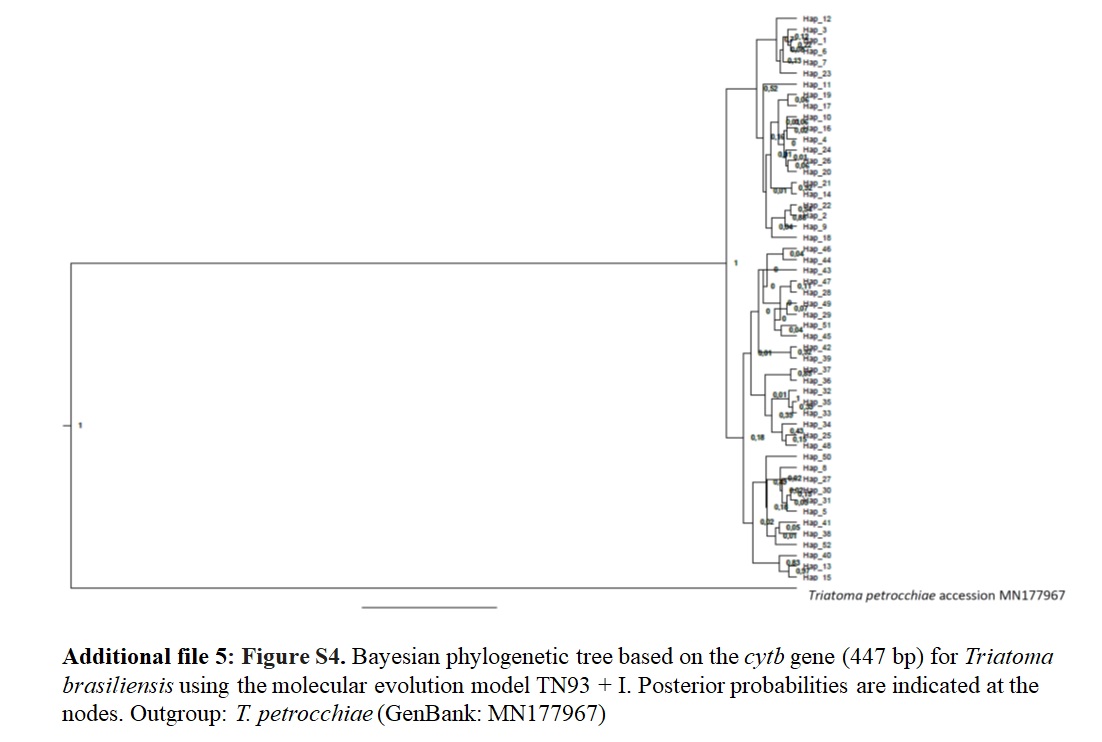

Supplement: Supplementary file 5 — Additional file 5: Figure S4. Bayesian phylogenetic tree based on the cytb gene (447 bp) for T. brasiliensis using the molecular evolution model TN93 + I. Posterior probabilities are indicated at the nodes. Outgroup: T. petrocchiae (GenBank: MN177967). [file 13071_2020_4340_MOESM5_ESM.jpg]
